# The effects and safety of anticoagulation or antiplatelet therapy following TIPS in cirrhotic patients with portal hypertension: A meta-analysis

**DOI:** 10.3389/fphar.2023.1116177

**Published:** 2023-02-20

**Authors:** Xiaotong Xu, Yunlai Fu, Minjie Jiang, Muchen Wu, Jing Wu, Qinghua Meng

**Affiliations:** ^1^ Department of Oncology, Beijing You An Hospital, Capital Medical University, Beijing, China; ^2^ Department of Hepatology, Beijing You An Hospital, Capital Medical University, Beijing, China

**Keywords:** liver cirrhosis, hypertension, portal, portasystemic shunt, transjugular intrahepatic anticoagulants, platelet aggregation inhibitors

## Abstract

**Introduction:** Transjugular intrahepatic portosystemic shunt (TIPS) is an effective way to improve portal hypertension, however, the role of anticoagulation or antiplatelet therapy following TIPS remains controversial. We conducted this study to evaluate the efficacy and safety of anticoagulation or antiplatelet therapy following TIPS.

**Methods:** A literature search was conducted on anticoagulation or antiplatelet therapy after TIPS using Pubmed, Web of Science, EMBASE, and Cochrane. The retrieval period was from the earliest accessible date in the database to 31 October 2022. We collected information on the incidence of stent dysfunction, bleeding, hepatic encephalopathy, the new occurrence of portal vein thrombosis, and the survival rate. Stata was analyzed in RevMan.

**Results:** 1. Four studies received anticoagulation or antiplatelet therapy after TIPS without control groups. According to the single-group rate meta-analysis, stent dysfunction occurred at 27% [95% CI (0.19, 0.38)], bleeding occurred at 21% [95% CI (0.14, 0.29)], new portal vein thrombosis occurred at 17% [(95%CI(0.04.0.71)], hepatic encephalopathy occurred at 47% [95%CI (0.34, 0.63)], and death occurred at 31% [95% CI (0.22, 0.42)]. 2. Eight studies, including 1025 patients, compared anticoagulation and antiplatelet therapy after TIPS to TIPS alone. In terms of stent dysfunction, bleeding, and hepatic encephalopathy, there were no significant differences between the two groups. The use of anticoagulation or antiplatelet therapy may result in a significant decrease in the incidence of new portal vein thrombosis and mortality over 1 year.

**Discussion:** Anticoagulant or antiplatelet therapy may not improve the patency rate of TIPS, but may effectively prevent new portal vein thrombosis after TIPS. Following TIPS, the use of anticoagulants or antiplatelet drugs does not lead to an increase in bleeding or death.

## Introduction

Portal hypertension is a clinical syndrome characterized by gastrointestinal hemorrhage and refractory ascites resulting from an increase in pressure within the portal vein system. Liver cirrhosis is one of the most common causes of portal hypertension. Transjugular intrahepatic portosystemic shunt (TIPS) can be performed (Sankar and Moore, 2017) to mitigate portal hypertension and reduce the occurrence of the above adverse events. As the operation will damage blood vessels and liver tissues, and the stent is implanted as a foreign body, there is a risk of stent stenosis, blockage, and thrombosis in the stent (Cura et al., 2008). After stent implantation, patients with coronary heart disease require anticoagulation and antiplatelet treatment to prevent thrombosis in the stent (Shang et al., 2019). However, it remains controversial whether anticoagulation or antiplatelet treatment after TIPS may also reduce postoperative complications. Due to the fragile balance between the coagulation and anticoagulation systems of liver cirrhosis patients (Lisman et al., 2021), as well as the risk of bleeding, there have been few studies on anticoagulation or antiplatelet treatment after TIPS. At present, there is no clear consensus regarding whether anticoagulation or antiplatelet treatment is needed after TIPS (Boyer et al., 2010), and only limited studies have been conducted in this area. Therefore, we aimed to conduct a meta-analysis of existing studies to evaluate the efficacy and safety of anticoagulation or antiplatelet therapy after TIPS and provide a reference for clinical treatment.

## Materials and methods

**Search strategy:** With free words and subject words, we searched Pubmed, Web of Science, EMBASE, and Cochrane. Medical Subject Headings included “liver cirrhosis”, “hypertension, portal”, “portasystemic shunt, transjugular intrahepatic”, “anticoagulants” and “platelet aggregation inhibitors”. In the entry terms, “hepatic cirrhosis”, “portal hypertension”, “TIPS”, “anticoagulation agents”, “antiplatelet agents”, and so on were included. Except for “anticoagulants” and “platelet aggregation inhibitors” which were combined with Boolean logic symbols “OR”, all other search terms were combined with Boolean logic symbols “AND”. Data were retrieved from the earliest accessible date in the database until 31 October 2022. References were also screened. A manual search was conducted in addition to the electronic search to identify additional studies that were not found through the electronic search.

**Inclusion criteria:** A cohort of patients with liver cirrhosis and portal hypertension was included in the study. Study findings included at least one aspect of the following information regarding the use of anticoagulant or antiplatelet drugs after TIPS: stent dysfunction (stenosis, occlusion), new portal vein thrombosis, bleeding, hepatic encephalopathy, and death.

**Exclusion criteria:** Repetitive literatures, case reports, letters, reviews, and meta-analyses were excluded.

**FIGURE 1:**
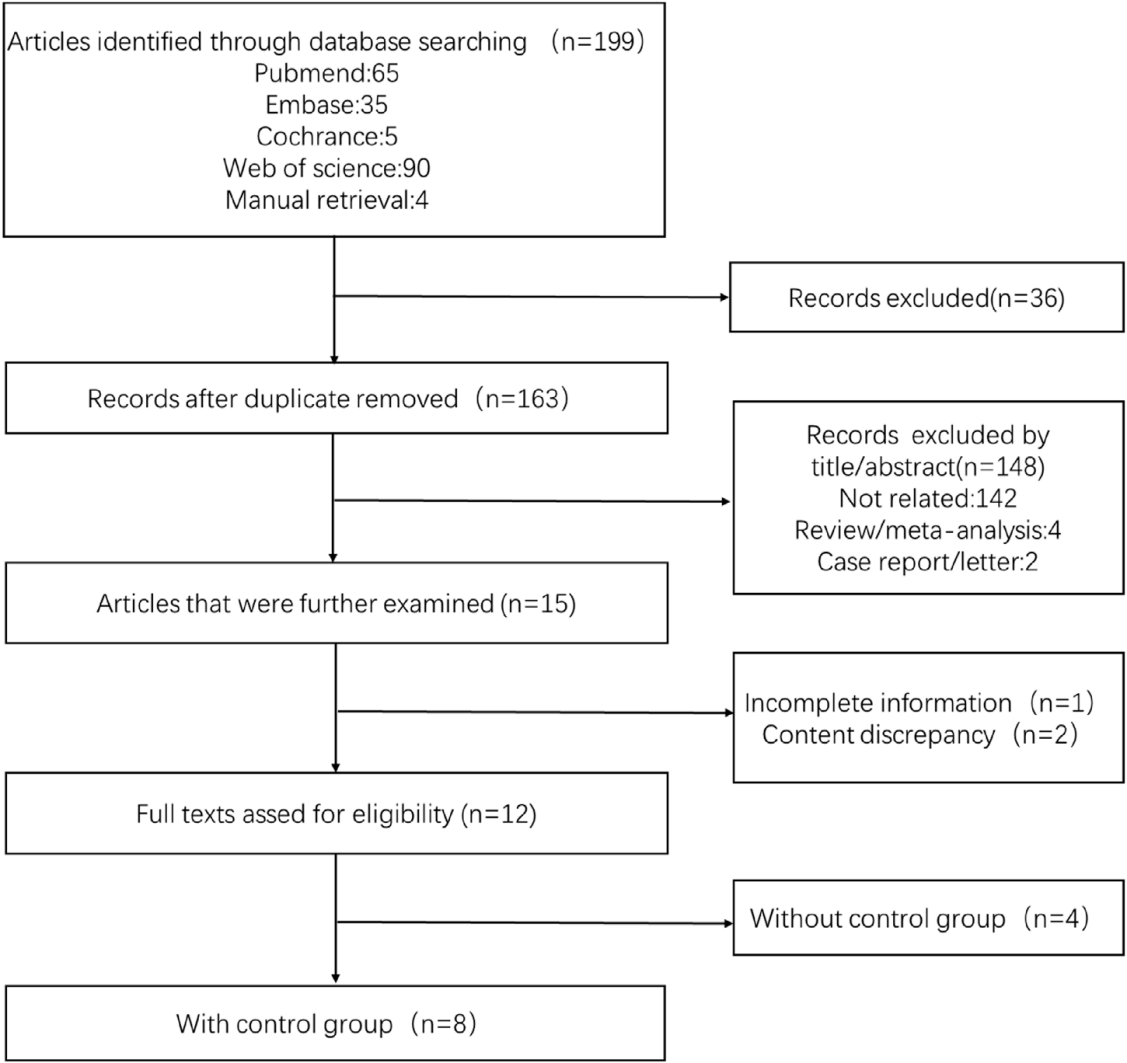
Flowchart of screening literature.

**Literature screening and quality evaluation:** We selected the literature that met the standards based on screening the titles and abstracts by two independent evaluators (Xiaotong Xu and Yunlai Fu). A quality evaluation table was used to evaluate the quality of the literature. In the randomized controlled study, the Cochrane risk bias evaluation tool was used, while in the observational study, the Agency for Healthcare Research and Quality (AHRQ) scoring table was utilized. For the ambiguous part, we would resolve it through discussion with a third evaluator. The process of filtering was illustrated in Figure 1. The information regarding the article’s quality evaluation was presented in Table 1.

**TABLE 1:** Quality assessment for studies.

The cochrane Collaboration’s tool for assessing randomized controlled trials															
Author/year/country		Selection bias			Performance bias		Detection bias		Attrition bias			Reporting bias		Other bias	
Theilmann et al. (1994) Germany		Low risk			Low risk		Low risk		Low risk			Low risk		Low risk	
Sauer et al. (1996) Germany		Low risk			Low risk		Low risk		Low risk			Low risk		Low risk	
Siegerstetter et al. (1997) Germany		Low risk			Low risk		Low risk		Low risk			Low risk		Low risk	
Wang et al. (2016) China		Low risk			Low risk		Low risk		Low risk			Low risk		Low risk	
Lv et al. (2018) China		Low risk			Low risk		Low risk		Low risk			Low risk		Low risk	
**The Agency for Healthcare Research and Quality scoring table for cohort studies**															
**Author/year/country**	**Representativeness of the exposed cohort**		**Selection of the non-exposed cohort**	**Ascertainment of exposure**		**Demonstration that outcome of interest was not present at the start of the study**		**Comparability between exposed and non-exposed cohort**		**Assessment of outcome**	**Was follow-up long enough for outcomes to occur**		**Adequacy of follow-up of cohorts**		**Total** **Score**
Tang et al. (2017) China	1		1	1		1		0		1	1		1		7
Zhang et al. (2020) China	1		1	1		1		1		1	1		1		8
Lv et al. (2021) China	1		1	1		1		2		1	1		1		9
Han et al. (2011) China	1		1	1		1		0		1	1		1		7
Wang et al. (2015) China	1		1	1		1		0		1	1		1		7
Wan et al. (2017) China	1		1	1		1		0		1	1		1		7
Seifert et al. (2022) Germany	1		1	1		1		2		1	1		1		9

**Data extraction and statistical analysis:** The following data was collected from the literature that met the inclusion and exclusion criteria: author, country, year, research type, the incidence of stent dysfunction, new portal vein thrombosis (PVT), hemorrhage, and hepatic encephalopathy (HE). After TIPS, the experimental group received anticoagulation or antiplatelet therapy, whereas the control group was not given anticoagulation or antiplatelet therapy. Considering the limited literature retrieved, we performed a single-group rate meta-analysis of the literature that does not include a control group. The collected data were analyzed in RevMan software, and the random effect model was selected.

## Results

Initially, 199 articles were searched in the database. A total of 12 articles were evaluated after screening. Finally, eight articles including 1025 patients were analyzed, of which 628 patients received anticoagulation or antiplatelet therapy after TIPS, while 397 patients received TIPS only. In addition, four of the studies included only the anticoagulation or antiplatelet treatment group following TIPS without a control group. Since there was a limited amount of related research, a single-group rate meta-analysis was conducted. Therefore, the results mainly included two aspects. The basic characteristics of the included articles were shown in Table 2. This analysis focused on stent function, bleeding, new occurrence of PVT, HE, and survival after TIPS.

**TABLE 2:** Basic characteristics of included studies.

Author/year/country	Study design	Stent type	Experimental VS. control group	Anticoagulation time
Theilmann et al. (1994) Germany	Randomized controlled study	Palma stent	Aspirin (*n* = 21) VS. Control (n = 21)	3 months
Sauer et al. (1996) Germany	Randomized controlled study	Palma stent	Phenprocoumon (n = 22) VS. Control (*n* = 23)	3 months
Siegerstetter et al. (1997) Germany	Randomized controlled study	Wallstent、Palmazstent	Heparin (n = 12) VS. Control (n = 12)	1 month
Wang et al. (2016) China	Randomized controlled study	Covered stent	Warfarin (n = 31) VS. Control (n = 33)	12 months
Tang et al. (2017) China	Cohort study	Covered stent	Aspirin (n = 95)/Clopidogrel (n = 64)/Warfarin (n = 9) VS. Control (n = 14)	6 months
Zhang et al. (2020) China	Cohort study	Covered stent	Warfarin (n = 27) VS. Control (n = 56)	12–33 months
Lv et al. (2021) China	Cohort study	Covered stent	Warfarin/Heparin/Rivarsaban (n = 197) VS. Control (n = 88)	21 months
Seifert et al. (2022) Germany	Cohort study	covered stents	Asprin (n = 150) VS. Control (n = 150)	Not mentioned
Han et al. (2011) China	Cohort study	Bare stent	Heparin-Warfarin-Aspirin (n = 43)	6 months
Wang et al. (2015) China	Cohort study	Covered stent	Warfarin (n = 25)	6 months
Lv et al. (2018) China	Randomized controlled study	Covered stent	Warfarin (n = 24)	9.3 months
Wan et al. (2017) China	Cohort study	Covered and bare stents	Warfarin (n = 52)/Aspirin, Clopidogrel (n = 74)	6 months

**FIGURE 2:**
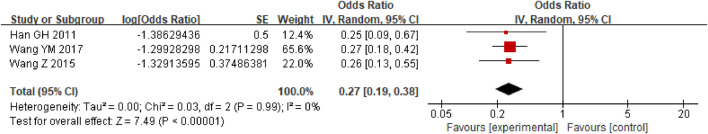
The occurrence of stent dysfunction.

**FIGURE 3:**
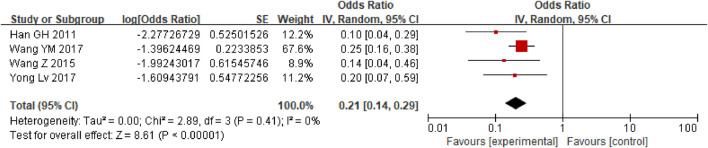
The occurrence of bleeding.

**FIGURE 4:**
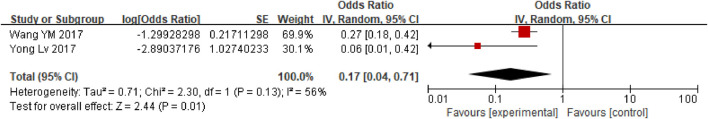
The new occurrence of portal vein thrombosis.

**FIGURE 5:**
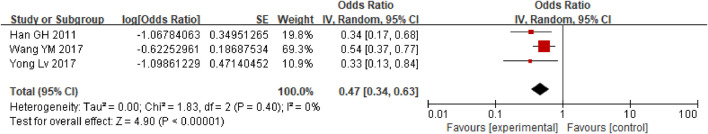
The occurrence of hepatic encephalopathy.

**FIGURE 6:**
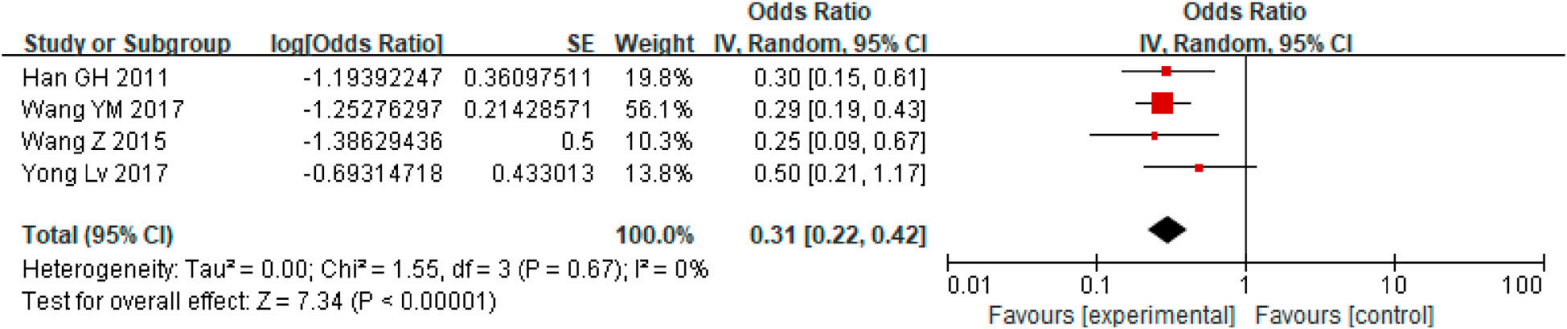
The occurrence of death.

First, a single-group rate meta-analysis of four studies was conducted in which only patients receiving anticoagulation or antiplatelet therapy after TIPS were included. The analysis was conducted based on the incidence of stent dysfunction, new occurrence of PVT, hemorrhage, HE, and death. Specifically, as shown in Figure 2, the results showed a 27% incidence of stent dysfunction [95% CI (0.19, 0.38)] in patients receiving anticoagulation or antiplatelet therapy following TIPS. The incidence of bleeding, new occurrence of PVT, HE, and death was 21% [95% CI(0.14, 0.29)], 17% [95% CI (0.04, 0.71)], 47% [95% CI (0.34, 0.63)], 31% [95% CI (0.22, 0.42)] respectively, as shown in Figures 3–6.

Moreover, eight studies were analyzed to compare the differences in stent dysfunction, bleeding, new occurrence of PVT, HE, and death between patients who received anticoagulation or antiplatelet therapy after TIPS, and those who received only TIPS.

### Stent dysfunction

**FIGURE 7:**
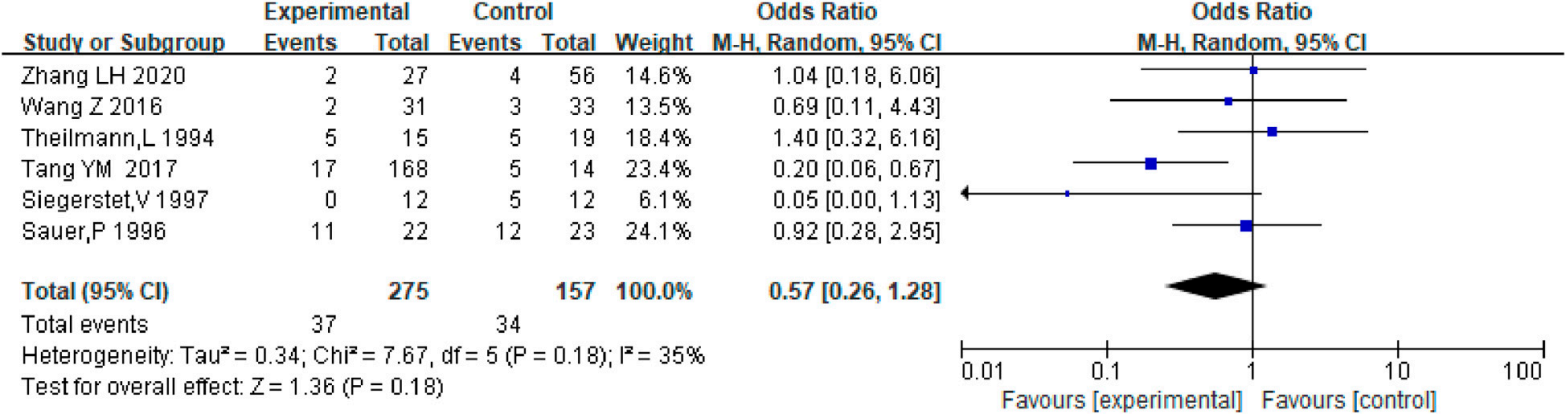
Effect of anticoagulation or antiplatelet treatment on stent dysfunction after TIPS.

Stent dysfunction includes stent stenosis and occlusion. Six studies including the data on stent dysfunction in patients receiving anticoagulation or antiplatelet therapy after TIPS and TIPS only were analyzed, and there was no significant difference between the two groups with respect to the incidence of stent dysfunction [OR = 0.57.95% CI (0.26–1.28), *p* = 0.18] as shown in Figure 7. And we analyzed the data of anticoagulation only after TIPS, with the similar results [OR = 0.56.95% CI (0.22–1.46), *p* = 0.24], as shown in Supplementary Figure S1. Considering the different types of stents and PVT, we further conducted subgroup analysis, as shown in Supplementary Figure S2–S4.

### Hemorrhage

**FIGURE 8:**
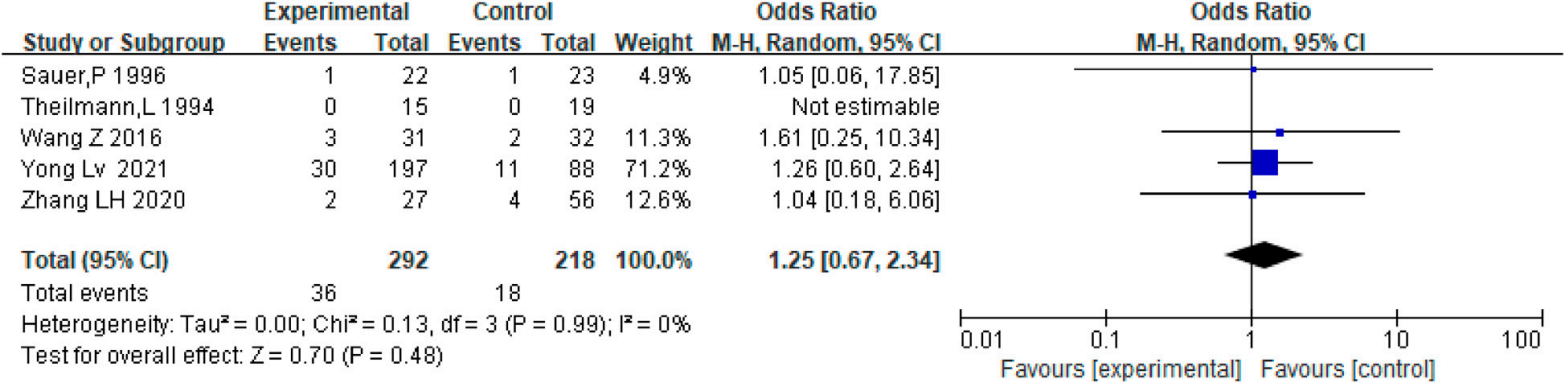
Effect of anticoagulation or antiplatelet treatment on bleeding after TIPS.

Hemorrhage mainly refers to major bleeding events, including gastrointestinal bleeding, intracranial hemorrhage, and hematuria. There were five studies, including the data on bleeding in patients who received anticoagulation or antiplatelet therapy after TIPS and TIPS only. Results show that there was no significant difference between the two groups regarding bleeding rates [OR = 1.25, 95% CI (0.67–2.34), *p* = 0.48], as shown in Figure 8. Considering the different types of stents, we conducted subgroup analysis, as shown in Supplementary Figure S5–S6.

### New occurrence of portal vein thrombosis

**FIGURE 9:**
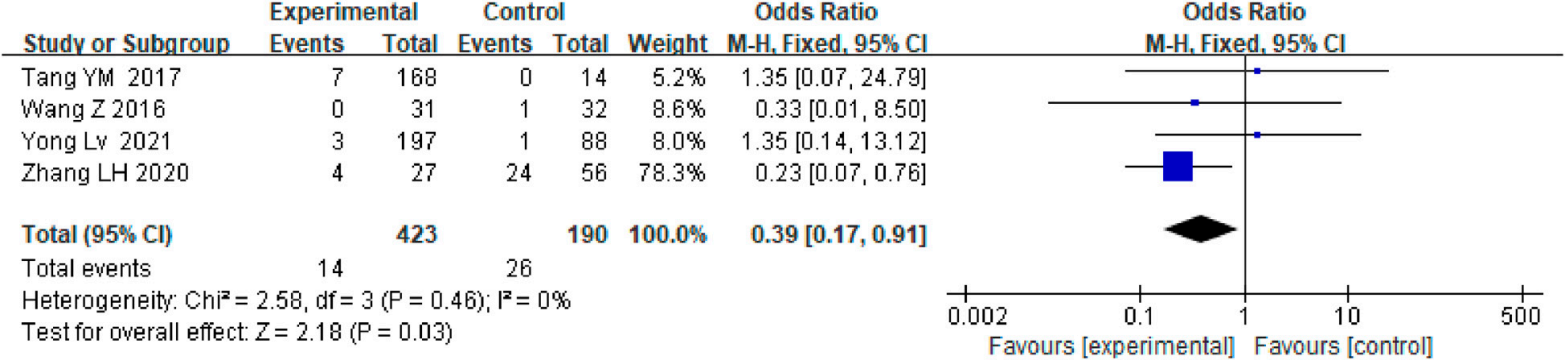
Effect of anticoagulation or antiplatelet treatment on new portal vein thrombosis after TIPS.

Anticoagulation or antiplatelet therapy after TIPS can significantly reduce the incidence of PVT [OR = 0.39.95% CI (0.17–0.91), *p* = 0.03] compared with patients who only underwent TIPS, as shown in Figure 9. The stents were all covered stents. Then, we further conducted subgroup analysis based on whether the subjects had PVT in the past, as shown in Supplementary Figure S7–S8.

### Hepatic encephalopathy

**FIGURE 10:**
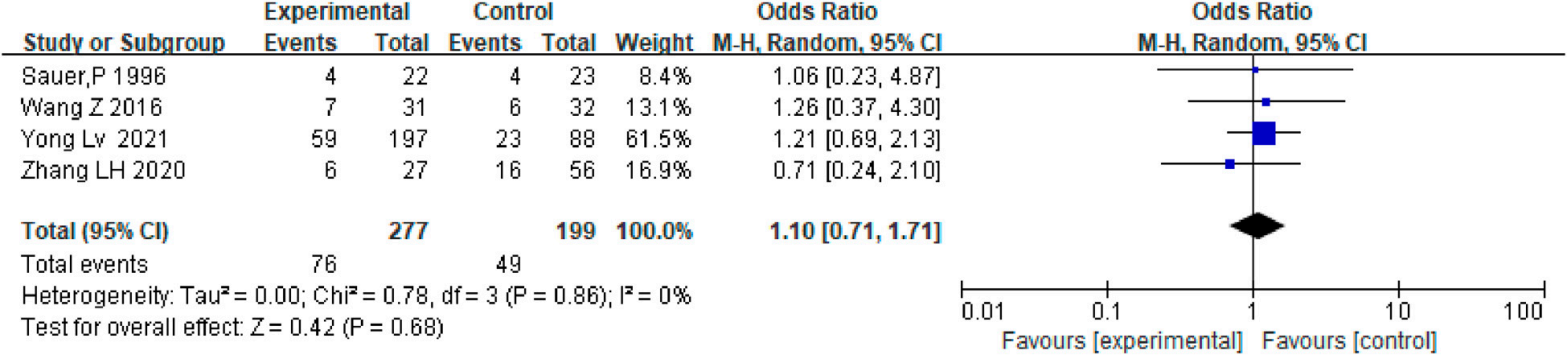
Effect of anticoagulation or antiplatelet treatment on hepatic encephalopathy after TIPS.

The incidence of HE did not differ significantly between patients who received anticoagulation and antiplatelet therapy after TIPS and those who only received TIPS [OR = 1.10.95% CI (0.71–1.71), *p* = 0.68], as shown in Figure 10.

### Occurrence of death

**FIGURE 11:**
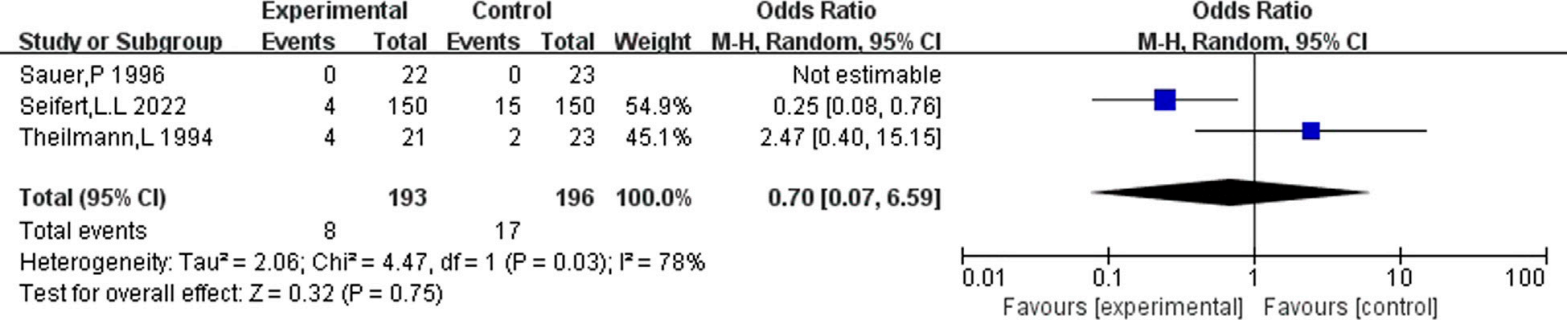
Effect of anticoagulation or antiplatelet treatment on 3 months death after TIPS.

**FIGURE 12:**
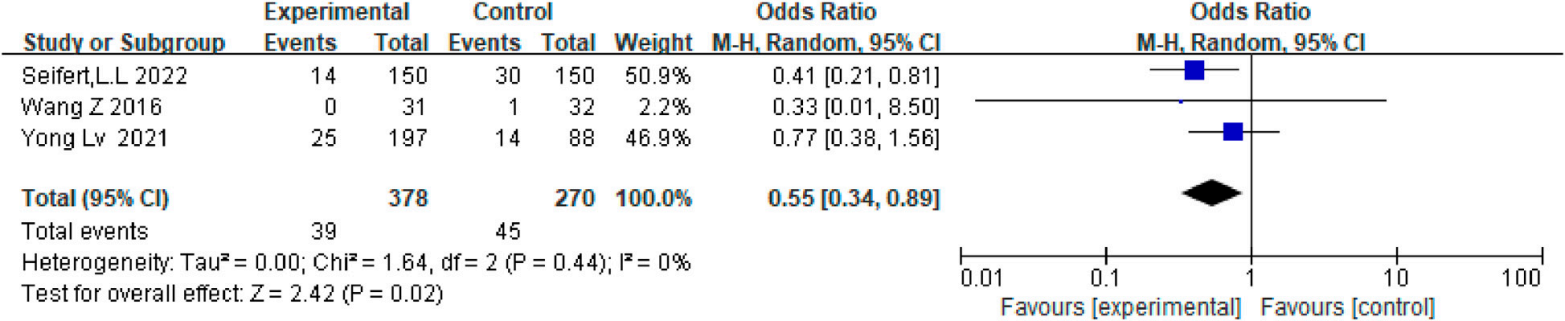
Effect of anticoagulation or antiplatelet treatment on 1 year death after TIPS.

**FIGURE 13:**
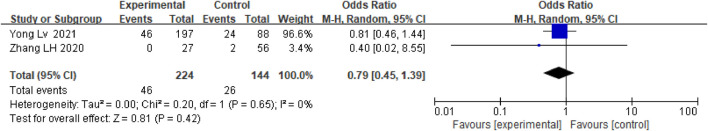
Effect of anticoagulation or antiplatelet treatment on 3 years death after TIPS.

Depending on the follow-up time, the data were analyzed for 3 months, 1 year, and 3 years. There was no difference in mortality between the two groups at 3 months (OR = 0.70.95%CI (0.07–6.59), *p* = 0.75) and 3 years [OR = 0.79.95%CI (0.45.1.39), *p* = 0.42], but anticoagulation or antiplatelet therapy after TIPS reduced mortality at 1 year [OR = 0.55.95%CI (0.34.0.89), *p* = 0.02], as shown in Figures 11–13.

## Discussion

In order to understand the incidence of stent dysfunction, bleeding and other events during anticoagulation or antiplatelet therapy after TIPS, we first conducted a single-group rate meta-analysis of four articles without control. In previous studies (Luca et al., 2011), dysfunction of bare stents in 1 year after TIPS was approximately 38% and that of covered stents was approximately 21%. In our study, anticoagulation or antiplatelet therapy after TIPS, there were approximately 27% of patients with stent dysfunction, 21% with bleeding,17% with new PVT,47% with HE and 31% death. It was concluded that the preventive use of anticoagulant or antiplatelet drugs after TIPS has limited effect on the reduction of stent dysfunction in patients undergoing TIPS.

Although TIPS is an effective treatment for portal hypertension, some complications may occur such as stent dysfunction (de Franchis and Baveno, 2015; Boike et al., 2022). The prognosis and survival may be affected by these adverse events (Dissegna et al., 2019). Based on the time of occurrence, stenosis after TIPS can be divided into early, middle, and late stages. An early stent stenosis occlusion is primarily caused by thrombotic stenosis. In the middle and long term, stent stenosis is mainly caused by excessive hyperplasia of pseudointima (Liu et al., 2021). Theoretically, postoperative anticoagulation or antiplatelet therapy can inhibit platelet aggregation and change the coagulation state, which is related not only to patients with PVT before TIPS (Dong et al., 2021), but also to the occurrence of possible stent stenosis or blockage after TIPS.

The evaluation of safety and effectiveness mainly included five aspects: bleeding, the new occurrence of PVT, HE, stent dysfunction, and survival. In our study, anticoagulation or antiplatelet therapy after TIPS did not reduce the incidence of stent dysfunction. In previous studies, bare stents were mostly used, and in current studies, covered stents are mostly recommended (de Franchis et al., 2022). Then we conducted subgroup analysis according to different stents. The result of our study was that whether using bare stents or covered stents, postoperative anticoagulation or antiplatelet therapy did not significantly reduce the incidence of stent dysfunction. The anticoagulant or antiplatelet drugs used in the study were inconsistent, but no further subgroup analysis was totally carried out due to the limited number of studies. We analyzed two articles of covered stents that only used warfarin, and the results were similar. There was no significant difference between the experiment group and the control group.

However, some studies have shown (Tang et al., 2017)that anticoagulation or antiplatelet therapy after TIPS can reduce the occurrence of stent dysfunction. According to Xia YF (Yifu et al., 2022), continuous anticoagulation after TIPS can effectively prevent stent stenosis or blockage within 30 days for patients with partial PVT before TIPS, but between 180 days and 90 days there was no significant difference. In patients with patency, spongiform change, or complete occlusion of the PVT, anticoagulation after TIPS had no significant impact on stenosis or occlusion of the stent. It should be noted that the study object was cirrhosis with PVT, so patients may be relatively hypercoagulable, and the use of anticoagulants reduced stent stenosis or obstruction. We tried to conduct a subgroup analysis based on whether there was PVT in the past and found that anticoagulation or antiplatelet therapy was ineffective in preventing the occurrence of stent dysfunction after TIPS.

In the past, TIPS was contraindicated for patients with PVT, but it is now being increasingly applied to cirrhosis patients with PVT. Researchers found that TIPS was as safe as anticoagulation and could reduce the load of PVT (Zhan et al., 2021). According to our research findings, the use of anticoagulation and antiplatelet therapy can prevent the occurrence of new PVT after TIPS. Following this, we conducted a subgroup analysis based on whether PVT had occurred in the past. Results showed that it could reduce the incidence of new PVT in patients without previous PVT. Therefore, it was effective for the *de novo* PVT, but not for the recurrence of PVT in patients with previous PVT. And Yong Lv (Lv et al., 2021) found that administration with Heparin or Rivalsaban after TIPS were associated with higher the probability of PVT recanalization, reduced thrombosis risk, improved survival rates. Angelo Luca (Wang et al., 2016) maintained that TIPS itself assisted in the recanalization of the PVT in patients with PVT by improving the flow of blood following the procedure, even in patients who are not prescribed anticoagulants or antiplatelet medications.

Anticoagulation or antiplatelet therapy after TIPS did not increase the risk of bleeding and HE, and the results were similar in subgroup analysis based on the different stents. Because almost all the included studies used warfarin, no further subgroup analysis was conducted.

In terms of survival, we found that anticoagulation or antiplatelet therapy after TIPS can reduce the 1-year mortality, but it did not significantly reduce the mortality of 3 months and 3 years after TIPS. The long-term effect of anticoagulation on the survival of TIPS still needs further study with large samples and randomized trial designs to draw firm conclusions.

According to a Germany survey on anticoagulant treatment following TIPS (Steib et al., 2020), 43 hospitals were included, four of which had not used an anticoagulant and eight of which had used both Aspirin and Clopidogrel. It is common for hospitals to use low-molecular-weight heparin for several days to 1 month. Due to the lack of clear guidelines, there are great differences in anticoagulation, antiplatelet therapy, and drug selection after TIPS (Fagiuoli et al., 2017; Rajesh et al., 2020). As there are few large-scale studies in this area and a limited amount of literature has been retrieved, further research is needed to evaluate the effectiveness of anticoagulation and antiplatelet therapy after TIPS.

Several systematic reviews or meta-analysis focusing on anticoagulation treatment after TIPS have been published recently. In the Guo et al. (2022) article, they only analyzed the incidence of bleeding based on the different anticoagulation percentages. But in our study, instead of analyzing the incidence of bleeding, we also compared the new occurrence of PVT, stent dysfunction, HE, and survival. Our research results were consistent with Jiao et al. (2022). Anticoagulation after TIPS can prevent the incidence of new occurrence of PVT. And we updated the included articles.

Some potential limitations of this study should be acknowledged. First, the number of relevant literatures retrieved was limited, and although the subjects included in the study were all patients with liver cirrhosis, some of them were accompanied by PVT, and the timing of thrombosis and the specific degree of blockage were not clear. Furthermore, the therapy option of anticoagulant and antiplatelet drugs were not consistent between different studies, which might also have a potential effect on final results. And current recommendations discourage (European Association for the Study of the Liver, 2022) the use of Vitamin-K-antagonists in patients with cirrhosis due to the unreliable results of coagulation monitoring. However, due to limited data, we cannot conduct subgroup analysis based on different anticoagulant drugs. Therefore, studies with more patients and longer follow-up time based on different anticoagulant or antiplatelet drugs should be carried out.

## Conclusion

Although anticoagulation or antiplatelet therapy after TIPS may not improve the short-term shunt patency rate, it may effectively prevent the formation of new portal vein thrombosis and decreased 1-year mortality. In terms of safety, the use of anticoagulants and antiplatelet drugs after surgery will not increase the incidence of bleeding and death.

## Data Availability

The original contributions presented in the study are included in the article/Supplementary Material, further inquiries can be directed to the corresponding author.
