# Supplementary material for: The effects and safety of anticoagulation or antiplatelet therapy following TIPS in cirrhotic patients with portal hypertension: A meta-analysis
Source: Front Pharmacol. 2023 Feb 20;14:1116177. doi: 10.3389/fphar.2023.1116177 (PMC9986321; doi:10.3389/fphar.2023.1116177)
Supplement: Supplementary file 1 [file Presentation1.pdf]

Supplementary Appendix

Study or Subgroup	Experimental Events	Experimental Total	Control Events	Control Total	Weight	Odds Ratio M-H, Random, 95% CI
Sauer,P 1996	11	22	12	23	39.2%	0.92 [0.28, 2.95]
Siegerstet,V 1997	0	12	5	12	8.9%	0.05 [0.00, 1.13]
Tang YM 2017	0	9	5	14	8.9%	0.09 [0.00, 1.88]
Wang Z 2016	2	31	3	33	20.6%	0.69 [0.11, 4.43]
Zhang LH 2020	2	27	4	56	22.4%	1.04 [0.18, 6.06]
**Total (95% CI)**		**101**		**138**	**100.0%**	**0.56 [0.22, 1.46]**
Total events	15		29			

Heterogeneity: $\text{Tau}^2 = 0.25$; $\text{Chi}^2 = 5.03$, df = 4 (P = 0.28); $I^2 = 20\%$
Test for overall effect: Z = 1.19 (P = 0.24)

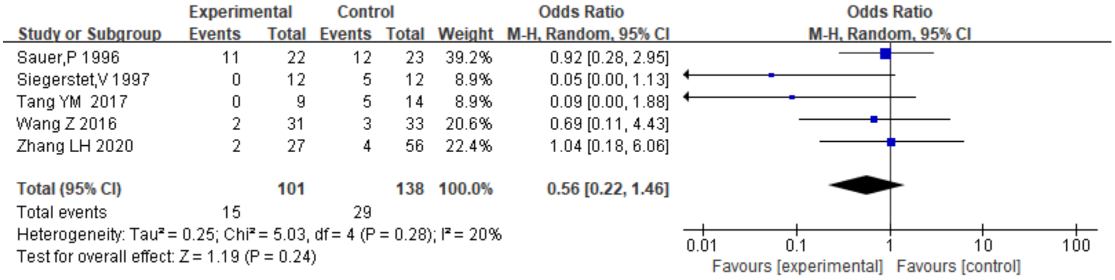

Figure S1. Effect of anticoagulation treatment on stent dysfunction after TIPS

Study or Subgroup	Experimental Events	Experimental Total	Control Events	Control Total	Weight	Risk Ratio M-H, Random, 95% CI
Sauer,P 1996	11	22	12	23	56.8%	0.96 [0.54, 1.70]
Siegerstet,V 1997	0	12	5	12	8.0%	0.09 [0.01, 1.48]
Theilmann,L 1994	5	15	5	19	35.2%	1.27 [0.45, 3.58]
**Total (95% CI)**		**49**		**54**	**100.0%**	**0.88 [0.38, 2.02]**
Total events	16		22			

Heterogeneity: $\text{Tau}^2 = 0.23$; $\text{Chi}^2 = 3.46$, df = 2 (P = 0.18); $I^2 = 42\%$
Test for overall effect: Z = 0.31 (P = 0.75)

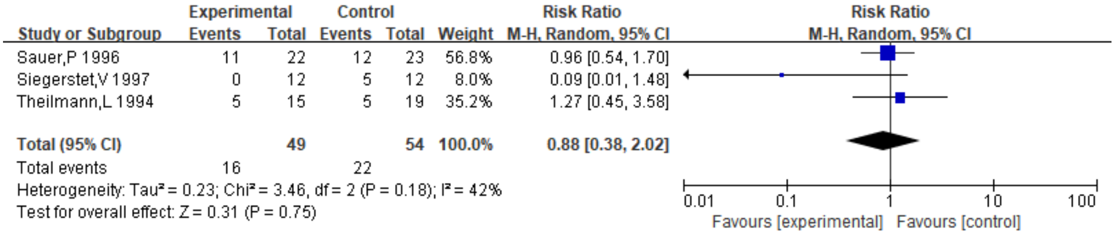

Figure S2. Effect of anticoagulation or antiplatelet treatment on stent dysfunction after TIPS (uncovered stents, without PVT previously)

Study or Subgroup	Experimental Events	Experimental Total	Control Events	Control Total	Weight	Risk Ratio M-H, Random, 95% CI
Tang YM 2017	17	168	5	14	56.1%	0.28 [0.12, 0.65]
Wang Z 2016	2	31	3	33	21.0%	0.71 [0.13, 3.97]
Zhang LH 2020	2	27	4	56	22.9%	1.04 [0.20, 5.31]
**Total (95% CI)**		**226**		**103**	**100.0%**	**0.46 [0.19, 1.11]**
Total events	21		12			

Heterogeneity: $\text{Tau}^2 = 0.17$; $\text{Chi}^2 = 2.71$, df = 2 (P = 0.26); $I^2 = 26\%$
Test for overall effect: Z = 1.73 (P = 0.08)

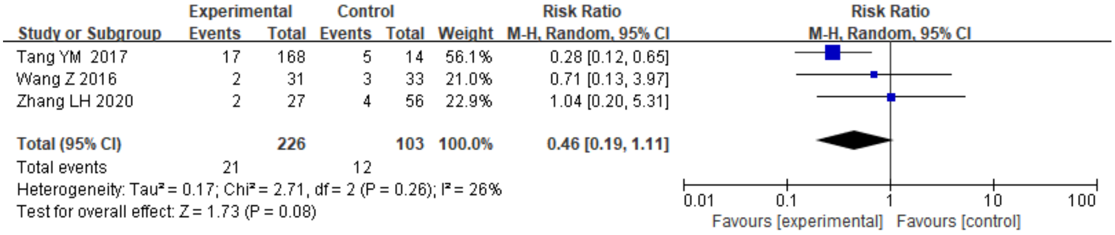

Figure S3. Effect of anticoagulation or antiplatelet treatment on stent dysfunction after TIPS (covered stents)

Study or Subgroup	Experimental Events	Experimental Total	Control Events	Control Total	Weight	Odds Ratio M-H, Random, 95% CI
Tang YM 2017	17	168	5	14	57.8%	0.20 [0.06, 0.67]
Zhang LH 2020	2	27	4	56	42.2%	1.04 [0.18, 6.06]
**Total (95% CI)**		**195**		**70**	**100.0%**	**0.40 [0.08, 2.02]**
Total events	19		9			

Heterogeneity: $\text{Tau}^2 = 0.79$; $\text{Chi}^2 = 2.33$, df = 1 (P = 0.13); $I^2 = 57\%$
Test for overall effect: Z = 1.10 (P = 0.27)

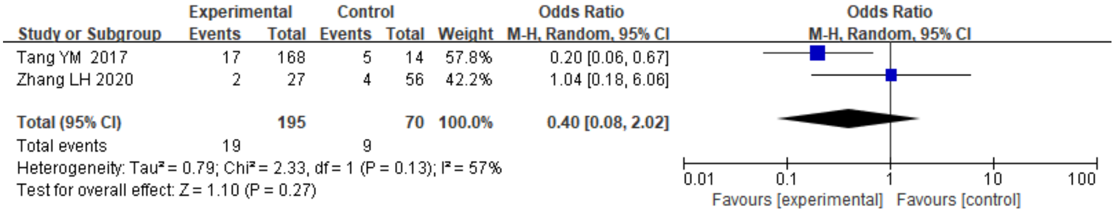

Figure S4. Effect of anticoagulation or antiplatelet treatment on stent dysfunction after TIPS (covered stent, without PVT previously)

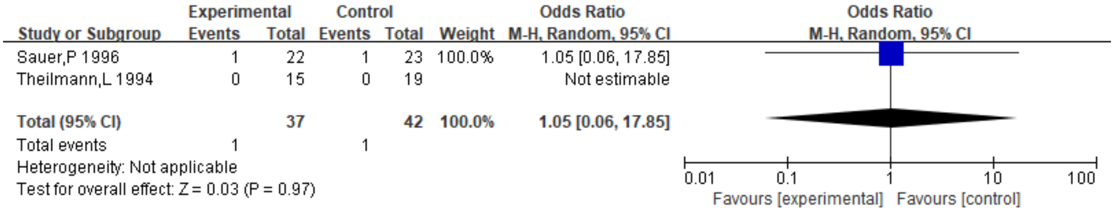

Figure S5. Effect of anticoagulation or antiplatelet treatment on bleeding after TIPS (uncovered stents)

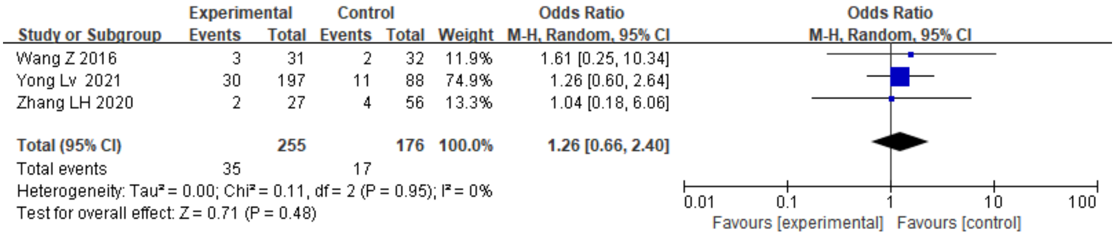

Figure S6. Effect of anticoagulation or antiplatelet treatment on bleeding after TIPS (covered stents)

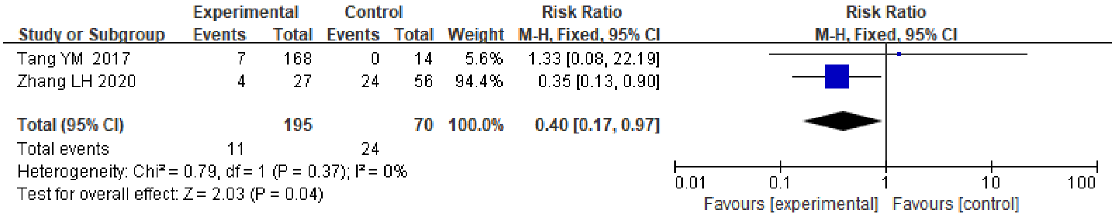

Figure S7. Effect of anticoagulation or antiplatelet treatment on new portal vein thrombosis after TIPS (without PVT before)

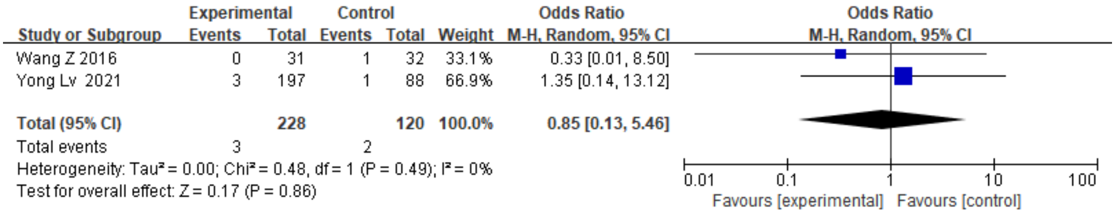

Figure S8. Effect of anticoagulation or antiplatelet treatment on new portal vein thrombosis after TIPS (with PVT before)
